# Editorial: Endoscopic spine surgery

**DOI:** 10.3389/fsurg.2022.1127851

**Published:** 2023-01-09

**Authors:** Yong Yu, Zhen-Zhou Li, Yasuhiko Nishimura

**Affiliations:** ^1^ Department of Neurosurgery, Zhongshan Hospital, Fudan University, Shanghai, China; ^2^ Department of Orthopaedic Surgery, The Fourth Medical Center of Chinese PLA General Hospital, Beijing, China; ^3^ Department of Neurosurgery, Wakayama KOYO Hospital, Wakayama, Japan

**Keywords:** endoscopic, spine, uni-portal, bi-portal, robot-assisted

**Editorial on the Research Topic** Endoscopic spine surgery

Endoscopic surgery has been widely accepted in the whole surgical field, as well as in the field of spinal surgery. The characteristic of endoscopic surgery is to puts the camera eye in the target area for close observation and projects the magnified high-definition image onto the screen. The surgeons look at the screen and perform the operation under the “indirect” visualization, which needs the training of hand-eye coordination. This feature is different from traditional surgery through loupe magnification or microscopic magnification (1). However, more and more surgeons, encouraged by the incomparable high-definition images, have devoted themselves to endoscopic surgery. I would like to make an analogy that an excellent endoscopic surgeon is like a master who is directing a high-definition blockbuster movie in the operating room.

Endoscopic spine surgery was initially used for lumbar disc lesions (2). However, it has evolved dramatically in recent years with the rapid development of endoscopic armamentaria and technological innovations, as well as a better understanding of endoscopic anatomy and approaches. As a result, the indications of endoscopic spine surgery are ever-expanding, from the initial lumbar disc degeneration to other types of pathologies located from craniovertebral junction to sacral vertebrae (3, 4).

In this research topic of Frontiers in Surgery, in the field of percutaneous single-channel endoscopic spine surgery, some attempts and efforts have been made to solve different pathologies. Ye et al. reported that they decompressed the medulla oblongata successfully using full-endoscopic uniportal retropharyngeal odontoidectomy (https://doi:%2010.3389/fsurg.2022.973064). There have been several papers focusing on this technique since Rutten first reported it in 2018 (5–7). As we all know, craniocervical junction pathologies are complex and challenging. In recent years, with the rapid development of posterior internal fixation and reduction technology, some patients do not need to undergo anterior odontoidectomy (8, 9). Therefore, the role of this technique in the whole treatment strategy to the craniocervical junction pathologies should be carefully considered and evaluated. Yu et al. reported a case with lumbar spinal epidural lipomatosis (SEL) who was treated with a percutaneous full-endoscopic uniportal decompression surgery successfully (https://doi.org/10.3389/fsurg.2022.894662). Percutaneous endoscopic surgery provides another option for SEL which has similar clinical symptoms to lumbar spinal stenosis. In addition, in terms of lumbar degenerative pathologies, Ahn et al. described a new surgical technique of endoscopic lumbar foraminotomy (ELF) for radiculopathy due to foraminal stenosis in patients with stable advanced spondylolisthesis, which the exiting nerve root be decompressed by resecting upper pedicle, lower vertebral endplates and SAP (https://doi.org/10.3389/fsurg.2022.1042184). This is a challenging technique, and the authors have obtained good results. It will be of interest to the readership of our topic research because of innovative thinking and technology, although there are some limitations such as lack of the control group, without long time follow-up and relative high complication rate.

Altogether twenty-two papers have been accepted due to its highlights in this topic research. It is worth noting that the number of papers focusing on Unilateral Biportal Endoscopy (UBE) technology is increasing. UBE technology has better freedom and compatibility with traditional surgery due to the separation of operation channel and observation channel. Some techniques are very interesting. Zhu et al. introduced a novel suture anchor techniques for cervical laminoplasty using UBE (https://doi.org/10.3389/fsurg.2022.913456), which shows in this “endoscopic dream factory” where everything is possible, even beyond all imagination. Of course, if a kind of technique can be popularized, it also needs other necessary conditions, such as definite safety, effectiveness, easy to learn and convenient tools.

Whether uniportal or biportal, literatures on endoscopic lumbar interbody fusion have grown tremendously in the last several years (10–12). Not surprisingly, the same is true of our research topic. there were five articles discussing this issue. This phenomenon indicates that the interest of endoscopic spine surgeons seems to have transited from simple decompression to further fusion after 20 years of full development. Lin et al. retrospectively compared the surgical outcomes between percutaneous endoscopic lumbar interbody fusion (PE-LIF) and minimally invasive transforaminal lumbar interbody fusion (MIS-TLIF) for the treatment of lumbar spinal stenosis. They concluded that both PE-LIF and MIS-TLIF are safe and effective for LSS. PE-LIF has a definite short-term curative effect with less trauma (https://doi.org/10.3389/fsurg.2022.916087). Endoscopic LIF may be the preferred options for select patients, such as the elderly. Techniques and instruments for endoscopic LIF have evolved over the past decade, leading to clinical and radiologic outcomes have improved, with particular benefits seen within ERAS pathways. However, just like any new technology, spine surgeons should be aware of the learning curve necessary before achieving operative mastery to minimize unique complications that can occur. At the same time, the fusion rate under water irrigation environment seems to need more convincing evidence.

Although endoscopic spine surgery has abundant merits that need not be detailed here, some obstacles make the learning curve steep and the surgical outcome is strongly dependent on the surgeon's practice of personal cultivation. These obstacles include confusing anatomical orientation, difficult to manipulate in a narrow space and so on. As a result, grafting new technologies such as navigation or robotics into endoscopic spine surgery has emerged (13). Ye et al. reported two cases of successfully treated lumber pyogenic spondylodiscitis using Da Vinci robot-assisted laparoscopic retroperitoneal approach (https://doi.org/10.3389/fsurg.2022.930536). The robot system provides high-definition images of three-dimensional vision and endo-wrist of the robot exceeds the limit of human hands which can perform precise movements continuously without fatigue and error during the procedure. There is no doubt that in the future, more new technologies, such as robots and intelligent navigation, will be integrated into endoscopic spinal surgery, which can bring revolutionary changes to spinal surgery.
